# Exploring the Experiences of Living With the Post‐COVID Syndrome: A Qualitative Study

**DOI:** 10.1111/hex.14108

**Published:** 2024-06-19

**Authors:** Michail Kalfas, Caroline Jolley, Nicholas Hart, Gerrard F. Rafferty, Emma L. Duncan, Timothy Nicholson, Mark Ashworth, Debbie Brewin, Barbara Barrett, Oliver C. Witard, Damien Ridge, Trudie Chalder

**Affiliations:** ^1^ Department of Psychological Medicine, Institute of Psychiatry, Psychology & Neuroscience King's College London London UK; ^2^ Centre for Human and Applied Physiological Sciences, King's College London Faculty of Life Sciences & Medicine London UK; ^3^ King's College Hospital NHS Foundation Trust London UK; ^4^ Lane Fox Clinical Respiratory Physiology Research Centre Guy's & St Thomas's NHS Foundation Trust London UK; ^5^ Department of Twin Research and Genetic Epidemiology King's College London London UK; ^6^ Guy's & St Thomas's NHS Foundation Trust London UK; ^7^ Institute of Psychiatry, Psychology, and Neuroscience King's College London London UK; ^8^ School of Life Course & Population Sciences, King's College London Guy's Campus London UK; ^9^ School of Social Sciences University of Westminster London UK

**Keywords:** COVID‐19, dismissal, gender, Long COVID, patient and public involvement, qualitative

## Abstract

**Introduction:**

Many people experience persistent symptoms for more than 12 weeks following SARS‐CoV‐2 infection, which is known as post‐COVID‐19 condition (PCS) or Long COVID (LC). PCS can impair people's quality of life and daily functioning. However, there is a lack of in‐depth research exploring the PCS patient journey, as well as gendered aspects of patients' experiences.

**Methods:**

Nineteen semi‐structured qualitative interviews were conducted with people living with PCS in the United Kingdom (13 women, 6 men). Interviews were transcribed verbatim and analysed inductively using reflexive thematic analysis.

**Results:**

Five main themes were identified: ‘Symptom dismissal’, ‘Lack of information and support’, ‘Life before and after Long COVID’, ‘Psychological impact’ and ‘Acceptance’. A shift overtime to self‐management of symptoms was evident. These themes represent different stages of patients' PCS journey. Narratives indicated that women highlighted dismissal by healthcare professionals (HCPs), which was not as prominent in men's narratives. In addition, women went into more detail about the psychological impact of PCS compared to men.

**Conclusion:**

Women with PCS reported symptom dismissal by HCPs, which may have delayed their diagnosis and negatively affected their well‐being. We were not able to explore the experiences of people from non‐conforming gender groups. Raising awareness of these issues among HCPs, particularly general practitioners, could improve patient care in PCS.

**Patient or Public Contribution:**

Patient and public involvement consisted of people who took part in the interviews and commented on the themes' interpretation and study conclusions.

## 1 Introduction

The post‐COVID syndrome (PCS) is described by the National Institute for Health and Care Excellence as the persistence of COVID‐19 symptoms for more than 12 weeks following primary infection, not explained by another diagnosis [1], known informally as ‘Long COVID’ (LC). Recent self‐reported estimates from the Office for National Statistics indicate that as of 5 March 2023, 1.9 million people in the United Kingdom reported experiencing ongoing symptoms for at least 12 weeks following SARS‐CoV‐2 infection [2].

People with PCS experience a constellation of symptoms, the most common of which are fatigue, dyspnoea, cognitive difficulties (often described as ‘brain fog’), reduced exercise capacity, post‐exertional malaise and muscle pains [2, 3]. Other PCS symptoms include heart palpitations, sleep disturbance, skin rashes and gastrointestinal symptoms [1]. These are shared with many other post‐acute infection syndromes [4]. Symptoms significantly impair people's quality of life and daily functioning [5, 6]. Accordingly, research indicates that 7 months following COVID‐19 infection, 45.2% of people required a reduced work schedule, and 22.3% were not working due to persistent symptoms [7].

The pathophysiology of PCS is not well understood. Suggested possible pathophysiological mechanisms include, but are not limited to, organ and tissue damage, immune dysregulation, dysautonomia, persistence of SARS‐CoV‐2 in certain tissues and chronic inflammation [8, 9, 10]. However, definitive evidence of these has not been established. Risk factors associated with PCS include high BMI, age, female sex, comorbidities and poor pre‐pandemic health [11, 12].

Currently, there are limited treatment options for PCS, and many people report feeling unsupported and isolated [13, 14]. Qualitative studies suggest that people with PCS experience difficulties accessing the National Health Service (NHS); and when they do, the care is considered inadequate [13, 15, 16]. In addition, experiences of symptom dismissal and unsatisfactory interactions with healthcare professionals (HCPs) are common among people with PCS [16, 17, 18]. As a result, affected individuals tend to self‐manage their symptoms or join online groups to seek validation and support [13, 19, 20].

It is yet to be explored whether the experiences of people living with PCS are gendered. Research in chronic pain indicates that there is a gender bias towards people with chronic pain, with women more often reporting symptom dismissal [21, 22]. PCS can be an ‘invisible’ condition whose diagnosis relies on self‐reported symptoms rather than medical tests. As a result, it is possible that women with LC also experience gender bias in healthcare. To our knowledge, no qualitative studies have specifically explored gendered dimensions of participants' narratives in PCS.

The primary objective of this qualitative study was to explore the experiences of people with PCS and assess whether these differed between those who identified as men or women.

## 2 Methods

### 2.1 Overview

This qualitative study was part of a project aiming to explore the experiences, needs and perspectives of people with PCS on a novel intervention that the study team was developing for PCS. The findings presented here represent only the first part of the qualitative interviews, which focused on the healthcare experiences and needs of people with PCS. The second part focused on the acceptability and feasibility of an intervention for PCS developed by the research team and will be presented in a separate paper. This project was conducted in the United Kingdom and received ethical approval (MRA‐21/22‐33455).

### 2.2 Participants

People who had given their permission to be contacted for future research in PCS were recruited. A few participants (*n* = 5) directly contacted the research team through word‐of‐mouth. Thirty participants were sent the participant information sheet, 19 of whom signed the consent form and took part in the interviews. The study researcher countersigned the consent form and sent it to participants via email.

### 2.3 Data Collection

Interviews were conducted remotely by M. K. between August 2022 and February 2023 on Zoom or Microsoft Teams. Interviews ranged from 26 to 80 min in duration, with an average duration of 45 min. Before the beginning of the interviews, participants verbally confirmed that they had signed the consent form and agreed to take part in the interviews. Participants were informed that they were not required to share sensitive information, they were free to skip any questions they did not want to answer, and were free to withdraw at any time without giving any reason. Interviews were semi‐structured and included open‐ended questions (see Table S1). Subsequent interviewer questions were guided by participants' responses (e.g., discussing acceptance). The interview guide included questions about participants' experiences of using healthcare services and having interventions for PCS. For example, participants were asked open‐ended questions such as ‘Can you please tell me about your experience of using NHS services for LC?’. The interview guide is presented in the appendix (Table S1). The term ‘Long COVID’ was used during these interviews, given widespread recognition and patient use of this term, rather than ‘PCS’.

### 2.4 Data Analysis

The qualitative interviews were transcribed verbatim and transcripts were imported on NVivo (version 12) software. Transcripts were thoroughly discussed within the research team. Reflexive thematic analysis was used inductively to analyse and interpret the data set. Reflexive thematic analysis involves the process of reflecting on the researcher's assumptions and subjectivities [23, 24]. Provisional codes were generated that were subsequently collated into initial themes by M. K. The initial themes, codes and their interpretation were thoroughly discussed with D. R. and T. C. and subsequently fine‐tuned. D. R., an expert in qualitative research, identified gendered differences in the narratives and suggested coding for—and exploring those—in subsequent analysis. T. C., an expert in cognitive behavioural therapies, highlighted the role of acceptance within the narratives. The similarities in the experiences of people with PCS and other long‐term health conditions, such as chronic pain and chronic fatigue syndrome (CFS), were also discussed. M. K. believed dismissal should be a central theme, as it was a highly prevalent and concerning finding, which has also been described in other conditions, such as chronic pain [21]. M. K. created a unifying model of the ‘Long COVID Journey’, based on the themes and narratives, which was refined by D. R. and T. C. Following this discussion, some themes were combined (e.g., ‘life before and after long COVID’ and ‘identity disruption’) and renamed. The final themes and draft manuscript were reviewed by all study members.

A patient and public involvement (PPI) group (*n* = 4; 3 women, mean age = 51) comprised of people with PCS who took part in the interviews and were involved in the interpretation of findings. An online group meeting took place in which the themes and relevant quotations were presented to PPI members. This was followed by a more general discussion on the study findings, including gendered aspects of participants' experiences. PPI members were also asked whether the researchers had missed any important themes or misinterpreted their narratives.

## 3 Results

Table 1 outlines participants' demographic characteristics. Most participants identified as women (*n* = 13) and White British (*n* = 15). The key themes identified were: ‘Symptom dismissal’, ‘Lack of information and support’, ‘Life before and after LC’, ‘Psychological impact’ and ‘Acceptance’.

**Table 1:** Demographic characteristics of the study sample.

Participant number	Sex	Age	Ethnicity
P001	Female	50	White British
P002	Female	33	White British
P003	Female	35	White British
P004	Female	55	White British
P005	Female	38	White Other
P006	Female	51	Black British
P007	Female	61	White European
P008	Female	49	White British
P009	Female	30	White British
P010	Female	49	Other
P011	Female	47	White British
P012	Male	59	White British
P013	Female	75	White British
P014	Female	38	White British
P015	Male	78	White British
P016	Male	40	White British
P017	Male	62	White British
P018	Male	54	White British
P019	Male	46	White British

### 3.1 Symptom Dismissal

The disbelief of participant accounts by HCPs was a common experience for participants with PCS, with participants frequently reporting that their symptoms were dismissed. While regularly experiencing such dismissals from their general practitioners (GP), participants' symptoms were often disregarded by more than one kind of HCP. A woman (P008) stated that she understood that there were no clear answers for PCS, but she never expected to have to struggle for her PCS recognition. Remarkably, one man described that he was not only dismissed but also ridiculed by his HCP:

I always accepted I had an illness that they had no answers for and no solutions for, but what I hadn't expected to have to fight to be believed.

(P008, woman)

I had an experience with a guy…he was basically, he dismissed me, he was rude, he was arrogant, and he told me to join the gym which obviously didn't help me in any way or form…he laid back in his chair and laughed at me.

(P018, man)

There was a gendered dimension to the narratives, with symptom dismissal frequently highlighted in accounts of participants identified as women, with only one man identifying the problem (P018). Nevertheless, only five participants identified as men were included in the interviews. Most women made a connection between their gender and their symptom dismissal. Men appeared to be taken more seriously by HCPs, whereas symptoms described by women were attributed to other factors, such as stress. Although women stated they felt more commonly dismissed by man HCPs, symptom dismissal by women HCPs was also reported:

I saw one doctor who told me well, you know, sometimes women choose to stay sick because they don't want to go back to the pressures of their everyday lives and we have to recognise that us women, we just have a lot more pressure in our lives, the expectations laid upon us are huge.

(P005, woman)

Participants acknowledged that the dismissal they experienced from HCPs had significant psychological and healthcare impacts, with implications for their PCS management. In most cases, people with PCS could only be referred to secondary services for PCS through their GPs; and therefore, if GPs dismissed them, they could not access NHS care. In addition, these negative experiences created a sense of mistrust and disappointment towards HCPs, which deterred people from visiting their GP in the future. This might have negatively affected people's symptom trajectory and quality of life. Participants often concluded that self‐managing their symptoms was easier than seeking medical advice (see first quote). The only man reporting dismissal described how the experience caused him distress and demoralisation:

I didn't go to the GP for years despite having constant symptoms. I just dealt with it by myself because it was easier…I wasn't having to deal with people saying that I was just making things up.

(P002, woman)

And he put me on a big downer, a very big downer to the point, you know, I was thinking well there's no point in going on now.

(P018, man)

### 3.2 Lack of Information and Support

Most participants mentioned a general lack of information about PCS, including among HCPs. This observation was especially the case for those who developed PCS early on. Participants reported that this lack of information and support was frustrating and served to increase illness uncertainty:

That has been frustrating not to have actual solid information to go on.

(P001, woman)

It was really long wait of complete unknowns.

(P003, woman)

Many participants with PCS felt unsupported, which, combined with the lack of available information, meant they had no other option than to self‐manage their symptoms with strategies they developed individually or read/discussed online:

I do feel now that so many of us are out here searching for our own solutions.

(P013, woman)

However, using the internet as the main source of information is not straightforward, as it often includes unreliable health‐related information [25].

### 3.3 Life Before and After Long COVID

About half of the participants vividly described the contrast between things they were able to do before and after LC, highlighting the functional limitations imposed by their condition. Here, people talked about how their lives were severely disrupted by PCS and conceptualised PCS as a life‐changing event. For example, participant 2 reported that she was used to having a very active life and had run regularly before PCS, which she could not do anymore:

I was running half marathons before I got long COVID. I can't run at all now. I'm not allowed to, because it makes my heart rate go way too high.

(P002, woman)

Yeah, it's been a tough time, very tough time. Yeah, my life is changed 100%.

(P015, man)

In many cases, such disruptions were associated with a change in identity, as participants were not able to do the things that previously supported their identity, such as being active or pursuing a career:

you're also adjusting your whole identity.

(P014, woman)

I was losing my career, I was losing everything.

(P005, woman)

Women participants often reported that functional limitations were especially challenging for them in terms of their caregiver roles and assumed social identities:

…so many women from mid 30s to mid 50s who will nine times out of ten be caregivers of children or parents, in some kind of caring industry, so teaching, nursing…at that age used to doing multiple things, now barely being able to do anything.

(P014, woman)

Therefore, women not only experienced dismissal from HCPs to a greater extent than men but also highlighted the pronounced impacts of PCS on their social identities.

### 3.4 Psychological Impact

Some participants reported that the functional limitations of PCS and changes in self‐identity took a significant psychological toll on them. Specifically, participants recognised how different their life was living with PCS, and highlighted the things they were not able to do anymore, and the links to psychological distress. Participants who identified as women talked more openly about their emotions, whereas men seemed less inclined to refer to the psychological impact of PCS in their narratives:

take anyone in society who's been fit and healthy training for a marathon, like me, and having run three marathons to go to not being able to get on the sofa and walk to the shop for 10 minutes…and ask yourself if that will make you feel quite depressed.

(P008, woman)

I'm so sick of being a sick person.

(P010, woman)

Many participants described feeling guilty about not being able to invest in their relationships as they had previously, including helping their partners with household tasks or participating in activities they used to do together. This theme was reported by both men and women, suggesting that men might be more willing to discuss emotions as they pertain to significant others, rather than their own inner life.

These feelings of guilt affected participants' interpersonal relationships and seem to have contributed to the psychological impact of PCS.

I didn't actually cook any meals for, at least the first year and a bit of having long COVID, my husband was doing it all and he was washing up, and the guilt I was feeling of, like not even being able to do the washing up.

(P002, woman)

…it affects our relationships, it affects us sexually, affects us in so many different ways.

(P017, man)

### 3.5 Acceptance

Participants often drew attention to the issue of acceptance during the interviews and specifically highlighted how difficult it was to come to terms with their new circumstances and accept their new limitations and selves. For many participants, this meant ‘giving up’ their old identities following their PCS diagnosis, which could be especially challenging. There were also differences in the way participants referred to this identity change; some described it as becoming a new version of themselves, whilst others focused on their disability:

I think the hardest thing for me and for others that I've spoken to is the acceptance that something is potentially changed as ongoing and that we need to find a new version of ourselves…we're giving up our old selves and having to become somebody completely new.

(P001, woman)

You're a different person and you have to accept that, well at this moment in time it may change, it may not, but you have to accept that you're disabled and that's quite difficult to do really.

(P012, man)

The interpretation of this transition into PCS affected acceptance, and some participants appeared to have a more positive explanation that could facilitate acceptance. It should be highlighted that acceptance is not a passive process of giving up or giving in to persistent symptoms, but is the active process of trying to remain open to—and managing—a difficult situation.

Some women mentioned the need to grieve for their old lives, emphasising the loss experienced with PCS (first quote). One participant conceptualised acceptance as the last stage of grief and highlighted its importance. More specifically, this participant recognised that grieving and finally accepting her ‘new self’ was emotionally helpful (second quote):

There's a bit of a grieving process going on for our old lives.

(P001, woman)

…it is like the grief process almost, you have to go through those steps…being able to kind of accept all this is where I work and how do I move forward has helped me.

(P011, woman)

### 3.6 The Long COVID Journey

Taken together, the key themes included in this paper can be viewed as snapshots of different stages of the LC journey. When asked about their experiences, participants frequently described the events in chronological order, starting from the emergence of symptoms and their first contact with HCPs to the identity disruption, psychological impact, and finding potential ways to mitigate them. Participants often recognised the close relationship between the different parts of the journey:

The thought that your life will not be your life and you have to adapt to this new normal and you're getting very little information through from anybody is quite scary.

(P002, woman)

### 3.7 PPI Reflection on Study Findings

**Figure 1:**
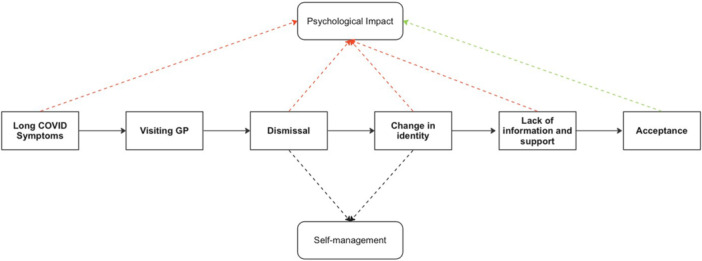
Stages of the patient journey and potential relationships between themes. Dashed red lines represent negative associations and green positive associations. Dashed black lines indicate ‘neutral’ associations, as the effectiveness of self‐management depends on the strategies used.

Feedback from the PPI group regarding study findings was affirming. All PPI members believed the themes were accurate and representative of their experience and were pleased with the schematic representation of ‘The long COVID journey’ (see Figure 1). The group also agreed that dismissal was gendered and that it would be important to raise awareness of this issue among HCPs and especially GPs. The group offered different perspectives on the research and wondered whether dismissal was more prevalent among those diagnosed at the beginning of the pandemic. Members also speculated on whether gendered differences could be attributed to the higher proportion of women in our sample. These points have been discussed in the discussion and limitation sections below.

## 4 Discussion

This study explored the experiences of people with PCS and highlighted gendered dimensions of living with the condition. Participants' responses suggested that the experience of dismissal by HCPs was a feature of women's narratives but less of an issue for men. Only gendered dimensions of people who identified as ‘men’ and ‘women’ were explored. Across England and Wales, a very small percentage of men (0.1%) and women (0.1%) identify as trans or nonbinary (0.1%) [26]. As qualitative research sampling does not aim to be representative [27], and our research was not devised to purposefully recruit people who were gender non‐conforming, all our participants identified as cis women and men. It will be important for future qualitative PCS research to be specifically designed and resourced to work with gender‐diverse participants to address this deficit. Narratives indicated that dismissal, combined with the lack of support, was associated with psychological distress and the move to self‐management of symptoms. Participants also described PCS as a life‐altering event, which had implications for their identity, with some recognising the importance of accepting their new (PCS) identities. Collectively, these themes seem to be interconnected and can be viewed as stages of patients' ‘Long COVID journey’.

Dismissal by HCPs was a highly prevalent theme across women's narratives, and women believed that they were dismissed due to their gender. Similar experiences of symptom dismissal have been reported by women with vulvodynia, endometriosis and chronic pain [21, 22, 28, 29]. It may be possible that men were taken more seriously by HCPs, whilst symptoms reported by women were attributed to psychological factors consistent with gendered norms in chronic pain [22]. These negative experiences were associated with feelings of distrust and frustration towards HCPs and led participants to attempt to self‐manage their symptoms, frequently with little guidance or support. This may negatively affect illness trajectory due to delayed treatment. More specifically, in most cases, people could only access secondary healthcare services for PCS through GP referrals. It is also possible that disbelief of participant accounts might have been more prevalent among those diagnosed at the beginning of the pandemic when PCS was not yet described, although this was not assessed in the present study.

Dismissal by HCPs has been reported by other qualitative studies in PCS and for CFS, but gendered dimensions were not examined and/or uncovered [16, 18, 30, 31]. However, Au et al. [17] qualitatively analysed surveys in PCS which indicated that women's responses about interactions with HCPs were more explicit and included more negative terms than men. This may suggest that women had more negative interactions with HCPs, or, alternatively, were more open to sharing these experiences than men. Nevertheless, some people with PCS report having positive interactions with HCPs [19].

Consistent with the study by Au et al. [17], our findings indicate differences in the way men and women describe their experiences and emotions. In our interviews, women talked more openly and in greater detail about the psychological impact of PCS compared to men. Men seemed to avoid revealing vulnerability during the interviews, which has also been described in other qualitative studies [32, 33]. This observation may be linked to social stereotypes of masculinity as involving stoicism [34]. It should be noted that all interviews were conducted by a man, and therefore, due to social norms, men might have been more reluctant to show vulnerability to another man. The influence of gender dynamics in qualitative interviews has also been described by other researchers [35, 36]. Interestingly, men seemed more willing to discuss more openly the impact of their condition on significant others and associated feelings of guilt towards partners, more so than the impact on themselves. Men might believe that expressing their emotions about significant others is more socially acceptable than describing their own inner psychological difficulties. Men's difficulty in talking about their mental health and experience of stigma is well documented in the literature [33, 37, 38].

Another prevalent theme was identity disruption following PCS diagnosis. Participants often highlighted the contrast between the activities they were able to do before PCS and their current physical functioning. In participants' narratives, PCS was portrayed as a highly disruptive life event that changed participants' identity and life trajectory. This theme resonates with the concept of ‘biographical disruption’, which was originally derived from qualitative interviews in rheumatoid arthritis. It refers to changes in the life trajectory and identity of people with long‐term health conditions [39]. Some participants described this change in identity as a transition to ‘a new version of their selves’, whereas others focused on the functional limitations of their new disabled selves. More specifically, women described grieving their old lives and identities. This observation highlights the magnitude of the loss in functioning experienced by people with PCS, as well as its implications for well‐being. This loss of identity has been found in other qualitative studies in PCS [15, 16, 40].

Acceptance was also discussed in the interviews. More specifically, some participants highlighted the need to accept their new lives and identities but also described acceptance as a highly active and demanding process. Acceptance was mostly described as part of the grieving process for their old lives. Acceptance is the final stage of grief, which, although it is not described necessarily as a ‘happy’ stage [41], has been associated with greater psychological quality of life and well‐being in people with chronic pain and spinal cord injury [42, 43]. This is in line with participants' narratives, which suggested that although acceptance was highly challenging, it was also beneficial. We should highlight, though, that acceptance was raised by the interviewer during the interviews, not conceptualised as a passive process of giving up, but rather as an active process of accommodating difficult situations and feelings and finding ways to manage them more effectively.

The themes identified in this study appear interrelated and describe the stages of participants' LC journey, as depicted in Figure 1. More specifically, the patient journey starts with the persistence of symptoms, compelling them to visit their GPs to seek advice and support. Subsequently, a significant proportion of these individuals, particularly women, were dismissed by their HCPs and consequently had to self‐manage their symptoms with little support or information. The persistence of PCS symptoms and functional limitations were associated with a change in their sense of self since they were no longer able to perform activities that supported their identity (e.g., exercising). All these stages and difficult experiences had a significant impact on psychological well‐being, as described in participants' narratives. Finally, participants highlighted the need to accept their new identity, which, although a challenging process, was described as emotionally helpful. As a note of caution, it should be highlighted that this schematic representation was based on the narratives of our sample and may not be representative for all people with PCS.

### 4.1 Strengths and Limitations

A strength of the present study is the comparison of narratives from men and women that highlight a gendered dimension of themes. In addition, our findings describe the relationship between the themes that are visually presented to illustrate the participants' LC journey. Finally, PPI members consisted of people who participated in the interviews and endorsed the interpretation of themes.

This study presents some limitations that warrant consideration. First, the study recruited a small sample of participants in the United Kingdom. More were women and White British, and unfortunately, we were not able to recruit more people from minority groups. As a result, our study sample is not designed to be representative of the wider patient population, and there are limits in generalising beyond White British patients. However, White women seem to represent the greater proportion of people with LC/PCS in the United Kingdom [11]. In addition, our sample is not representative of the full gender spectrum, as it only assessed the perspective of cis‐gendered people (i.e., identified as men/women) and did not include participants non‐conforming to the gender identities. The greater number of women in our sample also limited the comparisons we could make with men and the gendered conclusions we could draw. Nevertheless, we acknowledge that it is difficult to recruit men for qualitative research on chronic health conditions, and our efforts here led to valuable narratives from six men. In addition, although details on the PCS diagnosis timeline were not collected, narratives indicated that most participants developed PCS at the beginning of the pandemic, which might have implications for the generalisability of findings. In addition, we did not collect data regarding initial illness, PCS symptom severity and duration, as well as detailed sociodemographic characteristics (e.g., education level, employment status) which could have influenced participants' narratives.

### 4.2 Implications for Practice

Our study suggests that many people with PCS, particularly women, were often dismissed by their HCPs. These negative experiences were represented by greater distrust and frustration towards HCPs but increased symptom self‐management. Our findings are in line with research on women's health and chronic pain, which suggests that women commonly experience symptom dismissal, which might fuel feelings of perceived injustice. Therefore, a change in attitudes and clinical practice in relation to dealing with the so‐called ‘invisible illnesses’ is warranted. Our study aims to raise awareness of the negative patients' experiences among HCPs as these may influence future interactions with LC/PCS patients. It might be beneficial for HCPs to receive further training and education about PCS to facilitate understanding and provide better support to people with LC. Being able to conceptualise patients’ problems in such a way that conveys compassion and understanding cannot be underestimated. A formulation based on a concise summary of the origins and nature of a person's problems is the first step. An opinion on what steps might be taken to improve matters can then be offered up for discussion. This might include some information on what is known as well as what is not known about the post‐COVID symptom trajectory.

## 5 Conclusion

The present qualitative study adds to our understanding of the experiences of people with PCS and highlights gendered aspects of the narratives of cis‐gendered people with LC. The themes indicate that women experienced dismissal from HCPs to a greater extent than men, which is in line with gendered norms in chronic pain. This indicates that a change in the attitudes of HCPs as well as clinical practice in primary care is warranted. Participants also described the psychological impact of PCS more explicitly than men. The negative experiences of people with PCS may have delayed the diagnosis of PCS, which may have subsequently influenced the trajectory of the condition as well as interactions with HCPs. Unfortunately, our study was not resourced to recruit a more diverse sample on the whole gender spectrum, and therefore our results are only generalisable to those identified as cis‐gendered ‘men’ or ‘women’. Further research exploring the experiences of people with PCS/LC from minority and non‐conforming gender groups is needed.

## Author Contributions

**Michail Kalfas:** writing–original draft preparation, formal analysis; **Caroline Jolley:** conceptualisation, writing–review and editing, supervision. **Nicholas Hart:** funding acquisition, writing–review and editing. **Gerrard F. Rafferty:** writing–review and editing. **Emma L. Duncan:** writing–review and editing. **Timothy Nicholson:** writing–review and editing. **Mark Ashworth:** writing–review and editing. **Debbie Brewin:** writing–review and editing. **Barbara Barrett:** writing–review and editing. **Oliver C. Witard:** writing–review and editing. **Damien Ridge:** methodology, validation, writing–review and editing, conceptualisation, supervision. **Trudie Chalder:** supervision, investigation, funding acquisition, methods; writing–review & editing.

## Ethics Statement

This project received ethical approval (MRA‐21/22‐33455).

## Consent

Written informed consent was obtained from all participants.

## Conflicts of Interest

T.C. is part‐funded by the National Institute for Health Research (NIHR) Biomedical Research Centre at South London and Maudsley NHS Foundation Trust, King's College London. T.C. is the author of several self‐help books on chronic fatigue for which she has received royalties; has received ad hoc payments for workshops carried out in long‐term conditions and Long COVID; is on the Expert Advisory Panel for Covid‐19 Rapid Guidelines; has received travel expenses and accommodation costs of attending Conferences; is in receipt of other grants related to COVID from UKRI. D.R. has received funding from Roche for a project ‘Investigating how carers cope, access and use support services—Lessons from Covid‐19’, and is in receipt of another grant (NIHR) related to Long COVID. E.L.D. reports receiving funding for research into Long COVID from the Chronic Disease Research Foundation, specifically for the genetics of PCS/ongoing symptomatic COVID‐19. The remaining authors declare no conflicts of interest.

## Supporting information

Supporting information.

## Data Availability

The data are not publicly available as they contain private and sensitive information.
