# Supplementary material for: Exploring the Experiences of Living With the Post‐COVID Syndrome: A Qualitative Study
Source: Health Expect. 2024 Jun 19;27(3):e14108. doi: 10.1111/hex.14108 (PMC11186847; doi:10.1111/hex.14108)
Supplement: Supplementary file 1 — Supporting information. [file HEX-27-e14108-s001.docx]

**Supplementary Appendix**

**Table S1.** *Interview guide*

| **Topic** | **Questions** | **Prompts** |
| --- | --- | --- |
| **Experiences of using healthcare services for long COVID** | Can you please tell me about your experience of using NHS services for long COVID? |  |
|  |  | Were you happy with the care you received? |
|  |  | Did you have any bad experiences with NHS services? |
|  |  | Were the healthcare professionals understanding and knowledgeable about long COVID? |
|  |  | Did you experience dismissal from healthcare professionals? |
|  | Do you think your ethnicity, religion or gender, have influenced the care you have received? |  |
|  | *If yes, How so? Have you experienced any discrimination?* | *Did you feel you were treated with respect?* |
| **Experiences of receiving talking therapies** | Have you received any talking therapies? |  |
|  | *If yes*, did you find them helpful? What worked/did not work?  *If no,* have you received any other interventions? Did you find them helpful? Would you be willing to receive a talking therapy? |  |
| **Closing** | Is there anything else you would like to say? |  |
